# Endometrial Cancer in Young Women: Insights From a Tunisian Cohort

**DOI:** 10.7759/cureus.95784

**Published:** 2025-10-30

**Authors:** Mouna Kouira, Ekram Guerbej, Riadh Ncibi, Hafedh Abbassi, Chourouk Hafaiedh, Frigui Rym, Amal Aloui, Lassoued Latifa

**Affiliations:** 1 Department of Obstetrics and Gynecology, Faculty of Medicine "Ibn El Jazzar" of Sousse/Farhat Hached University Hospital, Sousse, TUN; 2 Department of Obstetrics and Gynecology, Higher School of Health Sciences and Techniques, Monastir, TUN; 3 Department of Health Policy, Higher School of Health Sciences and Techniques, Monastir, TUN

**Keywords:** central tunisia, endometrial cancer, fertility-sparing management, myometrial invasion, oncofertility, prognostic factors, survival outcomes, young women

## Abstract

Introduction

Endometrial cancer (EC) is typically diagnosed after menopause but can also occur in women under 40 years. In this younger subgroup, tumors are often low-grade and confined to the uterus, yielding favorable outcomes. The management of these patients raises the unique challenge of preserving fertility without compromising oncologic safety. Data from North Africa remain scarce.

Methods

We retrospectively reviewed medical records of women aged ≤40 years diagnosed with EC in the central region of Tunisia between January 2010 and December 2022. Clinical, pathological, imaging, and treatment data were analyzed. Statistical analyses were performed using IBM SPSS Statistics for Windows, Version 26.0 (Released 2019; IBM Corp., Armonk, New York, United States). Survival outcomes were estimated using the Kaplan-Meier method and compared with the log-rank test.

Results

Twenty-four patients were included, representing 6.3% of all 381 EC cases diagnosed at our institution between 2010 and 2022. The mean age was 34 years (range: 22-40). A history of infertility was reported in 19 of 24 patients (79.2%), preceding the diagnosis of cancer, and obesity was found in 14 (58.3%). Endometrioid adenocarcinoma was predominant in 16/24 (66.7%), followed by non-endometrioid histologies, including endometrial sarcoma (29.2%) and clear cell carcinoma (4.2%). Eight patients (33.3%) received fertility-sparing treatment consisting of oral high-dose progestins and/or a levonorgestrel-releasing intrauterine device. Adjuvant therapy, including radiotherapy, chemotherapy, or combined chemoradiotherapy, was administered to eight patients (33.3%), with no postoperative hormone therapy.

Conclusion

EC in women under 40 years is uncommon but generally associated with favorable outcomes. This study provides the first dedicated data on young women with EC from central Tunisia. Conventional pathological factors remain the main prognostic determinants in this resource-limited setting. Prospective studies incorporating molecular profiling are needed to refine risk stratification and guide tailored management strategies.

## Introduction

Endometrial cancer (EC), defined as malignant tumors arising from the endometrium, is the most common malignancy of the female reproductive tract and ranks among the most frequent cancers affecting women worldwide, with over 417,000 new cases and 97,000 deaths estimated in 2020 according to GLOBOCAN [1]. Its incidence is steadily rising in many regions, largely attributed to increasing life expectancy, obesity, and lifestyle changes [2]. Although EC is classically a postmenopausal disease with a median age at diagnosis of around 60-65 years [3], a proportion of cases occur in younger women.

Globally, women under 40 years represent approximately 4-7% of all EC diagnoses [4]. In this age group, the disease is often associated with obesity, nulliparity, chronic anovulation, polycystic ovary syndrome, infertility, diabetes, and hereditary cancer syndromes such as Lynch syndrome [5,6]. These risk factors reflect endocrine and metabolic disturbances that are well recognized in young women with EC. Pathologically, EC in young women is more frequently endometrioid type, low grade, and early stage at presentation [7], with a higher rate of synchronous ovarian cancer compared to older patients [8]. Prognosis is generally favorable due to early-stage, low-grade histology, although a subset can display aggressive behavior [9].

The management of EC in young women poses specific challenges, especially regarding fertility preservation. While the standard of care remains total hysterectomy with bilateral salpingo-oophorectomy (BSO)±lymph node assessment [3], this approach results in definitive sterility and premature menopause. Fertility-sparing protocols, generally based on high-dose progestin therapy, have shown promising oncologic and reproductive outcomes in carefully selected patients with early-stage, low-grade, endometrioid EC or atypical endometrial hyperplasia [10]. Ovarian preservation remains controversial; although it prevents the deleterious effects of surgical menopause, concerns persist about occult ovarian disease and synchronous malignancies [8,10].

In Tunisia, the most recent data from the Northern Tunisia Cancer Registry report EC as the third most common cancer among women, with an age-standardized incidence rate (ASR) of 9.3 per 100,000 [11]. Available epidemiological data confirm that young women represent a small but noteworthy subset of EC cases. However, there is a lack of updated data from the central regions, and no recent series has specifically addressed the clinicopathological characteristics of EC in women under 40 years. Our study will provide the most recent analysis of young women affected by EC in this region, aiming to adapt management strategies to local patient profiles and resources.

## Materials and methods

We conducted a retrospective review of medical records for all women aged ≤40 years diagnosed with EC, defined as malignant tumors arising from the endometrium, at the largest university hospital in the central region of Tunisia, Farhat Hached University Hospital, between January 2010 and December 2022. This study was performed in accordance with the Declaration of Helsinki and received approval from the Medical Ethics Committee of the Faculty of Medicine "Ibn El Jazzar" of Sousse (approval number: CEFMSo_0159_2025). Patients older than 40 years, those with incomplete data, or those who did not undergo surgical management were excluded.

Clinical and demographic data, including age, parity, comorbidities, and body mass index (BMI), were collected. Histopathological characteristics were obtained from surgical pathology reports and included tumor size, depth of myometrial invasion, lymphovascular space invasion, histologic subtype, tumor grade, cervical stromal invasion, and lymph node status. Staging was assigned postoperatively according to the 2009 International Federation of Gynecology and Obstetrics (FIGO) classification [12], and patients were reclassified according to the revised FIGO staging system [13] when data permitted.

The midline laparotomy was the most frequently used surgical approach, with systematic exploration of the abdomen and pelvis. Peritoneal cytology was not routinely performed. The standard surgical procedure consisted of total hysterectomy with bilateral salpingo-oophorectomy, combined with pelvic lymphadenectomy when stage and patient condition allowed. When pelvic lymphadenectomy was indicated, dissection was performed along the external and internal iliac vessels. Extension to the para-aortic area was carried out when indicated or depending on the surgeon's expertise and intraoperative assessment. Sentinel lymph node (SLN) mapping was not performed during the study period due to limited availability.

Histopathological analysis was conducted in accordance with the 2020 World Health Organization (WHO) classification of female genital tumors [14]. In our low-resource setting, immunohistochemical analyses were selectively performed when available, particularly for prognostic assessment in carcinoma cases, including microsatellite instability status and p53 expression. Molecular profiling of POLE exonuclease domain mutations was performed in a limited number of patients when cost coverage and resources permitted, using the Kompetitive Allele-Specific PCR (KASP) technique targeting five pathogenic "hot-spot" variants (S459F, V411L, P286R, S297F, and A456P) [15].

Adjuvant therapy (radiotherapy, chemotherapy, or combined treatment) was administered according to stage, nodal involvement, histopathological risk factors, and patient comorbidities. Follow-up visits were scheduled every three months during the first two years, every six months for the subsequent three years, and annually thereafter. Each visit included clinical and gynecological examinations, vaginal cytology, and pelvic ultrasound. Computed tomography or magnetic resonance imaging was performed annually or earlier if clinically indicated.

Progression-free survival (PFS) was defined as the time from primary surgery to recurrence or last follow-up. Overall survival (OS) was defined as the time from primary surgery to death or last contact. Statistical analysis was performed using IBM SPSS Statistics for Windows, Version 26.0 (Released 2019; IBM Corp., Armonk, New York, United States). Normality was assessed using the Shapiro-Wilk test. Continuous variables were analyzed with the Mann-Whitney U test, while categorical variables were compared with the chi-squared test. Survival outcomes were estimated using the Kaplan-Meier method and compared with the log-rank test. A p-value of <0.05 was considered statistically significant.

## Results

Clinical and epidemiological characteristics

A total of 24 women aged ≤40 years were diagnosed with EC between January 2010 and December 2022, representing 6.3% of all cases diagnosed at our institution between 2010 and 2022. The mean age at diagnosis was 34 years (range: 22-40).

A history of infertility was reported in 19 patients (79.2%), including 10 with primary infertility. Obesity (BMI ≥30) was present in 14 patients (58.3%), and overweight (BMI 25-29.9) in six patients (25%). The mean interval between symptom onset and first consultation was 16 months (range: 10 days to two years). The most common presenting complaint was infertility (41.7%), followed by pelvic pain (37.5%), menorrhagia (25%), and metrorrhagia (12.5%). Two patients presented with altered general condition.

Baseline demographic, clinical, imaging, histologic, and staging characteristics are summarized in Table 1.

**Table 1:** Baseline clinicopathologic and imaging characteristics of the study cohort (n=24) *Complex hyperplasia: recorded on biopsy but excluded from the analytic cohort if confirmed in the final pathology results and not associated with endometrial cancer BMI: body mass index; CBE: curettage and biopsy of the endometrium; MRI: magnetic resonance imaging; FIGO: International Federation of Gynecology and Obstetrics

Variable	Category	N	%
Age (years)	<34	14	58.3
	34-40	10	41.7
Personal medical history	None	17	70.8
	Hypertension	1	4.2
	Hematologic malignancy	1	4.2
	Anemia	1	4.2
	Epilepsy	1	4.2
	Hypothyroidism	1	4.2
Age at menarche	≤13 years	16	66.7
	>13 years	8	33.3
BMI (kg/m²)	Normal (19-25)	4	16.7
	Overweight (25-30)	6	25
	Obese (>30)	14	58.3
Circumstances of diagnosis	Infertility with desire for pregnancy	10	41.7
	Pelvic pain	9	37.5
	Menorrhagia	6	25
	Perimenopausal metrorrhagia	3	12.5
	General health deterioration (fatigue, weight loss, or anemia)	2	8.3
	Leukorrhea	1	4.2
	Hydrorrhea	1	4.2
Ultrasound findings	Thickened endometrium	15	62.5
	Intracavitary image	9	37.5
	Enlarged uterus	15	62.5
	Douglas pouch effusion	4	16.7
	Lateral uterine mass	3	12.5
Endometrial biopsy (CBE)	Endometrioid adenocarcinoma	15	62.5
	Complex hyperplasia*	1	4.2
	Endometrial stromal sarcoma	6	25
	Clear cell adenocarcinoma	1	4.2
	Choriocarcinoma	1	4.2
Grade of endometrioid adenocarcinomas	Grade I	6	40
	Grade II	6	40
	Grade III	3	20
MRI findings	Tumor limited to the endometrium	5	25
	Myometrial invasion <50%	6	30
	Myometrial invasion >50%	2	10
	Cervical involvement	2	10
	Adnexal involvement	1	5
	Vaginal involvement	2	10
	Pelvic lymph node involvement	2	10
				FIGO stage	IA	11	55
IB	2	10		
II	2	10		
IIIA	1	5		
IIIB	2	10		
IIIC1	2	10		
Management	Conservative treatment only	2	8.33
	Conservative treatment and then surgery	3	12.5
	Surgery only	5	20.83
	Surgery+adjuvant therapy	11	45.83
	Palliative chemotherapy	3	12.5
Final histopathological results	Endometrioid adenocarcinoma	16	66.66
	Undifferentiated endometrial sarcoma	7	29.16
	Clear cell carcinoma	1	4.16
Grade (endometrioid)	Grade I	7	43.75
	Grade II	6	37.5
	Grade III	3	18.75
Myometrial invasion	< ½	15	78.94
	> ½	4	21.05
Lymph node involvement	Yes	2	22.22
	No	7	77.77

Histopathological findings

All patients underwent an endometrial biopsy prior to treatment. On preoperative sampling, the most frequent histologic type was endometrioid adenocarcinoma (62.5%), followed by leiomyosarcoma (25%), clear cell carcinoma (4.2%), choriocarcinoma (4.2%), and atypical complex hyperplasia (4.2%).

Final surgical pathology confirmed the predominance of endometrioid adenocarcinoma (66.6%), leiomyosarcoma (29.1%), and clear cell carcinoma (4.2%). Endometrioid carcinomas were grade I in 68.7%, grade II in 18.7%, and grade III in 12.5%. Myometrial invasion <50% was observed in 70.8%, while 13.3% had nodal involvement. Surgical margins were negative in all cases.

FIGO 2009 stages were stage I (62.5%), stage II (25%), and stage III (12.5%). Immunohistochemistry and limited molecular testing (mismatch repair (MMR), p53, and POLE hot-spot variants) were performed in four patients. These cases represent the only subset in which additional analyses were undertaken, reflecting resource limitations rather than predefined selection criteria. Two of them had a microsatellite-stable (MSS) phenotype with normal p53 expression (Table 2).

**Table 2:** Characteristics of the four patients with available immunohistochemistry and molecular testing *: MSS (microsatellite stable); **: p53 WT (wild-type expression) MMR: mismatch repair; FIGO: International Federation of Gynecology and Obstetrics

Patient	Age	Personal history of neoplasia	MMR status	P53 expression	Histological type	FIGO grade	Treatment	Outcome
									P1	35	None	MSS*	WT**	Endometrioid adenocarcinoma	I	Conservative treatment and then extended colpohysterectomy	Alive in complete remission
P2	34	Previous history of ovarian cancer	Not performed	Not performed	Endometrioid adenocarcinoma	I	Extended colpohysterectomy+adjuvant chemotherapy	Alive in complete remission
P3	34	None	MSS	WT	Endometrioid adenocarcinoma	II	Extended colpohysterectomy+concomitant chemoradiotherapy	Alive with progressive disease
P4	34	None	Not performed	Not performed	Endometrioid adenocarcinoma	I	Extended colpohysterectomy	Alive in complete remission

Imaging and staging

Pelvic MRI was performed in 20 patients (83.3%). Reported findings included limited endometrial disease (25%), myometrial invasion <50% (30%) or ≥50% (10%), cervical invasion (10%), adnexal involvement (5%), vaginal involvement (10%), and suspicious pelvic nodes (10%). Preoperative FIGO distribution was IA (55%), IB (10%), II (10%), IIIA (5%), IIIB (10%), and IIIC1 (10%).

Treatment patterns

Nineteen patients (79.2%) underwent surgical treatment, of whom 16 (84.2%) received upfront surgery. Five patients (20.8%) received fertility-sparing conservative hormonal management with progestins and levonorgestrel-releasing intrauterine devices (IUDs); three of these eventually failed and subsequently required definitive surgery.

Peritoneal cytology was performed in 12 patients (50%) and was negative in 10. Lymphadenectomy was carried out in nine cases: five pelvic alone and four combined pelvic and para-aortic dissections. The mean number of nodes retrieved was 7 (range: 4-11).

The clinicopathologic characteristics and outcomes of the five women managed conservatively are summarized in Table 3.

**Table 3:** Characteristics and outcomes of patients initially managed with conservative treatment (n=5) FIGO: International Federation of Gynecology and Obstetrics

Case (n=5)	Age (years)	Histological type	FIGO stage	Outcome
C1	26	Endometrioid adenocarcinoma	IA	Under surveillance
C2	28	Endometrioid adenocarcinoma	IA	Under surveillance
C3	32	Endometrioid adenocarcinoma	IA	Failure→surgical management (extended colpohysterectomy without lymphadenectomy)
C4	34	Endometrioid adenocarcinoma	IA	Failure→surgical management (extended colpohysterectomy without lymphadenectomy)
C5	36	Endometrioid adenocarcinoma	II	Failure→surgical management (extended colpohysterectomy without lymphadenectomy)

Adjuvant therapy

Adjuvant treatment was indicated in 11 patients (45.8%): radiotherapy alone in four (36.4%), chemotherapy alone in four (36.4%), and combined chemoradiotherapy in three (27.3%). No patient received postoperative hormonal therapy (Table 4).

**Table 4:** Distribution of patients according to the therapeutic approach adopted (n=24)

Therapeutic approach	Number (n=24)	Percentage (%)
Surgery alone	5	20.8
Surgery+adjuvant treatment	11	45.8
Postoperative radiotherapy alone	4	36.4 of adjuvant cases
Postoperative chemotherapy alone	4	36.4 of adjuvant cases
Concomitant chemoradiotherapy	3	27.3 of adjuvant cases
Postoperative hormone therapy	0	0
Palliative chemotherapy	3	12.5
Conservative treatment alone	2	8.3
Conservative treatment followed by surgery	3	12.5

Oncologic outcomes

The mean follow-up duration was 20 months (range: 5-65). Fourteen patients were alive in complete remission at the last contact. Two patients developed local pelvic recurrences (mean interval: 11 months), both managed surgically with adjuvant therapy. Two patients developed pulmonary metastases (mean interval: 11 months), treated with chemotherapy. Two deaths were recorded, both attributable to disease progression in patients with sarcomatous histology.

Comparative statistical analysis

Comparisons of continuous variables between histological subgroups (Table 5) and associations of categorical clinicopathologic variables (Table 6) are shown. No significant differences were observed in age, BMI, or follow-up. Myometrial invasion ≥50% was more frequent in sarcoma/clear cell tumors, though this did not reach statistical significance.

**Table 5:** Comparison of continuous variables between histological groups (Mann-Whitney U test) BMI: body mass index

Variable	Endometrioid (n=16), median (IQR)	Sarcoma/clear cell (n=8), median (IQR)	Mann-Whitney U	P-value
Age (years)	34 (30-36)	35 (32-38)	51	0.42
BMI (kg/m²)	29 (27-32)	30 (28-33)	49	0.37
Follow-up (months)	21 (12-30)	19 (10-25)	54	0.55

**Table 6:** Association of categorical variables with histological type (chi-squared/Fisher's exact test) χ²: chi-squared test. Fisher's exact test was applied for variables with expected cell counts <5, specifically histologic subtype and distant metastasis, for which the χ² statistic is not applicable (N/A). An asterisk (*) indicates variables analyzed using Fisher's exact test. FIGO: International Federation of Gynecology and Obstetrics

Variable	Category	Recurrence (n, %)	No recurrence (n, %)	χ²	P-value
FIGO stage	I-II vs. III-IV	2 (10%) vs. 3 (75%)	18 (90%) vs. 1 (25%)	6.41	0.011
Histologic type	Endometrioid vs. non-endometrioid	2 (8.7%) vs. 3 (37.5%)	21 (91.3%) vs. 5 (62.5%)	N/A	0.046*
Myometrial invasion	<50% vs. ≥50%	1 (6.7%) vs. 4 (40%)	14 (93.3%) vs. 6 (60%)	5.92	0.015
Lymph node involvement	Negative vs. positive	1 (5.9%) vs. 2 (40%)	16 (94.1%) vs. 3 (60%)	2.14	0.16
Distant metastasis	Absent vs. present	2 (10%) vs. 2 (100%)	18 (90%) vs. 0 (0%)	N/A	0.045*

Survival analysis

**Figure 1:**
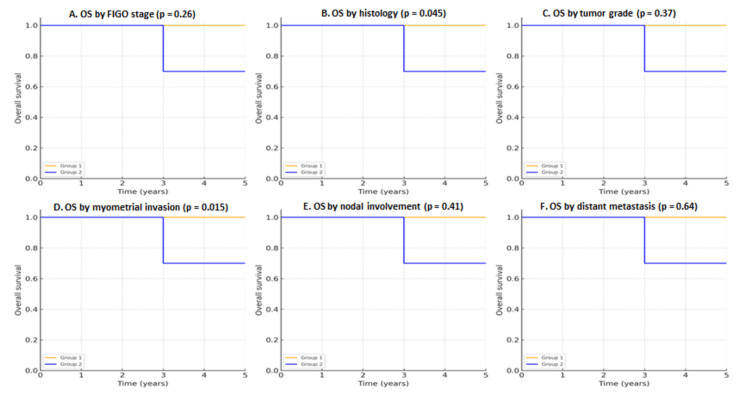
Kaplan-Meier OS curves in women ≤40 years with endometrial cancer, stratified by FIGO stage (A), histology (B), tumor grade (C), myometrial invasion (D), nodal involvement (E), and distant metastasis (F). Comparisons were performed using the log-rank test; corresponding p-values are indicated within each panel OS: overall survival; FIGO: International Federation of Gynecology and Obstetrics

The three- and five-year OS was 90.2%, with a mean survival time of 11.9 months (95% CI: 10.5-13.3). Survival was significantly influenced by histology, with OS rates of 100% in endometrioid adenocarcinoma compared to 71.1% in leiomyosarcoma (p=0.045). Tumor grade showed excellent OS for grade I and II tumors (100%), whereas grade III was associated with lower OS (71.4%), although the difference was not statistically significant (p=0.37). Myometrial invasion was a strong prognostic factor, with OS of 100% in patients with no invasion or <50% invasion versus 53.3% in those with ≥50% invasion (p=0.015). Node-negative patients had an OS of 86.8% compared with poorer outcomes in those with nodal involvement, though without statistical significance (p=0.41). The presence of distant metastases was associated with markedly reduced survival (50% at three and five years) (Figure 1).

## Discussion

In this study, we analyzed 24 cases of EC in women aged ≤40 years over a 12-year period at a tertiary referral center in central Tunisia. Young patients represented 6.3% of all EC cases, a proportion comparable to international reports that estimate 4-7% [4,7]. The clinical profile of this population has been well described, with strong associations with obesity, infertility, and endocrine disorders such as chronic anovulation and polycystic ovary syndrome [5,7]. Our findings are in line with these observations, particularly regarding the predominance of metabolic and reproductive risk factors in young women.

Histologically, endometrioid carcinoma is the most frequent subtype in this age group, usually low grade and confined to the uterus at diagnosis [7,9]. This explains the generally favorable prognosis compared with older women. In contrast, aggressive histologies such as clear cell carcinoma and endometrial stromal or undifferentiated sarcoma are uncommon in young patients [9,16]. The higher proportion of sarcomatous tumors in our series compared to previous reports warrants caution, as these histologies are associated with poor survival and remain a therapeutic challenge [16].

Prognostic determinants in young women with EC largely mirror those observed in the general population. FIGO stage, tumor grade, depth of myometrial invasion, lymphovascular space invasion, and nodal status have all been identified as predictors of recurrence and survival [3,9,17]. Our results confirm the prognostic weight of histology, myometrial invasion, and metastatic spread. Survival rates in our cohort were comparable to those reported in international studies, where five-year OS in young patients typically exceeds 85-90% [4,7,9].

The management of EC in young women raises specific concerns, particularly regarding fertility preservation, where hormonal therapy with high-dose progestins and levonorgestrel-releasing IUDs has shown acceptable oncologic outcomes in carefully selected early-stage, low-grade cases [10]. Reported complete response rates range from 55% to 80%, but recurrence is common, and strict follow-up with repeated endometrial sampling is essential [10]. Ovarian preservation also remains controversial: while it avoids the deleterious effects of surgical menopause, several studies report a risk of synchronous ovarian cancer in up to 5-29% of young EC patients [8,10]. These issues underline the need for individualized decision-making and long-term surveillance in fertility-sparing strategies.

Strengths and limitations

This study has several limitations, including its retrospective design, small sample size, relatively short follow-up, and restricted access to immunohistochemistry and molecular profiling. These factors limit the generalizability of our findings and the ability to classify tumors according to contemporary molecular risk groups. However, despite these limitations and the lack of routine molecular testing, our cohort demonstrated promising survival outcomes, largely determined by classical clinicopathological features, particularly histology and depth of myometrial invasion. A key strength of this study lies in its being, to our knowledge, the first dedicated series of young women with EC in central Tunisia, based on systematically collected data spanning more than a decade, thereby filling a regional gap in the literature.

## Conclusions

EC in women under 40 years is rare but generally associated with a favorable prognosis. Future prospective studies integrating molecular and immunohistochemical profiling are essential to refine prognostic assessment and guide management. Individualized strategies that balance oncologic safety with fertility preservation remain a cornerstone for this unique patient population.
